# HLA-check: evaluating HLA data from SNP information

**DOI:** 10.1186/s12859-017-1746-1

**Published:** 2017-07-11

**Authors:** Marc Jeanmougin, Josselin Noirel, Cédric Coulonges, Jean-François Zagury

**Affiliations:** Laboratoire Génomique, Bioinformatique et Applications, EA 4627, Conservatoire National des Arts et Métiers, 292 rue Saint-Martin, Paris, 75003 France

**Keywords:** Human leukocyte antigen, Major histocompatibility complex, Imputation

## Abstract

**Background:**

The major histocompatibility complex (MHC) region of the human genome, and specifically the human leukocyte antigen (HLA) genes, play a major role in numerous human diseases. With the recent progress of sequencing methods (eg, Next-Generation Sequencing, NGS), the accurate genotyping of this region has become possible but remains relatively costly. In order to obtain the HLA information for the millions of samples already genotyped by chips in the past ten years, efficient bioinformatics tools, such as SNP2HLA or HIBAG, have been developed that infer HLA information from the linkage disequilibrium existing between HLA alleles and SNP markers in the MHC region.

**Results:**

In this study, we first used ShapeIT and Impute2 to implement an imputation method akin to SNP2HLA and found a comparable quality of imputation on a European dataset. More importantly, we developed a new tool, HLA-check, that allows for the detection of aberrant HLA allele calling with regard to the SNP genotypes in the region. Adding this tool to the HLA imputation software increases dramatically their accuracy, especially for HLA class I genes.

**Conclusion:**

Overall, HLA-check was able to identify a limited number of implausible HLA typings (less than 10%) in a population, and these samples can then either be removed or be retyped by NGS for HLA association analysis.

## Background

Human Leukocyte Antigen (HLA) genes are coding for cell surface antigen proteins responsible for a major function of the immune system, the detection of foreign or abnormal antigens [1]. These genes are located on the short arm of chromosome 6, in a region known as the major histocompatibility complex (MHC). They play a ubiquitous role in medicine, most notably in autoimmune diseases [2, 3], infectious diseases [4, 5], and transplant medicine [6].

The MHC is among the most polymorphic regions in the human genome, with up to 4000 known alleles for each class I gene, and up to 2000 alleles for class II HLA genes (case of *HLA-DRB1*) [7]. Furthermore, there is a strong impact of natural selection in the evolutionary history of the MHC that creates long-range linkage disequilibrium observed between many if not most variants in this region [8], that further complicates the task of widely-used genomic tools such as imputation algorithms. Imputation algorithms typically use a reference panel to infer statistical patterns from linkage disequilibrium, that allows them to impute missing data in other datasets, usually using Hidden Markov Models on haplotypes [9].

The HLA typing technologies have evolved in the past few years from Sequence-Specific Primers (SSP) and Sequence-Specific Oligonucleotide Probes (SSOP) to Next-Generation Sequencing (NGS) [10]. SSP and SSOP were until fairly recently the best way to detect variations in the MHC but required known constant primers which could fail in the HLA region since some genes can have almost all of their nucleotides display polymorphisms (Single Nucleotide Polymorphism, hereafter SNP) [11]. These old methods also focused mostly on exons 2 and 3 for class I HLA genes (which code for the binding site), or just exon 2 in class II HLA genes, so many recorded HLA alleles are only known from these exons. NGS methods have now become robust enough to be used routinely [12], but are still too expensive for many research groups to afford: the order of magnitude of an HLA typing by sequencing is today 15 euros per allele typing, so 120 euros per individual for all 8 class I and class II loci. For panels consisting of thousands of people, this amounts to hundred of thousand euros for a typing by sequencing, while imputation methods and HLA-check allow to use already generated SNP data at no additional cost.

When typing HLA, the level of precision is usually called one-field (previously “2-digit”), two-field (or “4-digit”), or more. The first field indicates the serological antigen carried by an allotype, and the next ones the unique protein sequence. The next fields (not used in this study) indicate synonymous genetic polymorphisms. We’ll for instance denote an allele of the *HLA-A* gene at the one-field level as HLA-A*02 and at the two-field level as HLA-A*02:01.

Thanks to the availability of large reference panels being genotyped both by genotyping chips (Illumina, Affymetrix, other) and by NGS in the HLA region, several imputation methods have been developed in the past few years: SNP2HLA [13] (modeling HLA alleles as binary SNPs when running imputation software beagle), HIBAG [14] (R package using attribute bagging), or HLA*IMP [15] (Web service now discontinued). They exhibit a fairly good imputation accuracy level in the tests performed, ranging from 90 to 97% according to the HLA gene at stake [13]. Of course, this performance may greatly vary with the reference panel provided, as some studies have shown for instance that using a European panel for a Finnish population may lead to poor results [16]. It is also worth noting that these range of results do not allow any use of these methods in clinical settings, where the costs of HLA typing outlined above are minor compared to the medical consequences of a mistyping.

As discussed in previously published works, there are two important limitations for imputation methods: first, the diversity of the reference panel is crucial for the quality of imputation, and the possibility of errors in the reference panels due to failures in gold-standard typing methods may limit the imputation accuracy.

In the present work, we have developed a new tool which aims at limiting these sources of errors by evaluating the plausibility of the HLA alleles attributed to an individual given his SNP genotypes in the HLA region. With this tool, we could at the same time find errors in reference panels, and also evaluate the soundness of imputed HLA types obtained by any imputation tool. We show that we manage to drastically improve imputation, reaching 99% accuracy for some HLA genes while only eliminating a few individuals, and discuss the possible consequences of these observations.

## Implementation

### Data material

We primarily used the T1DGC (Type 1 Diabetes Genetics Consortium) cohort as our reference panel [17, 18] of 5225 European unrelated individuals. Genotype data included 7135 SNPs within the MHC region obtained with the Illumina Immunochip platform, and classical HLA allele typing for *HLA-A*, *HLA-B*, *HLA-C*, *HLA-DQA1*, *HLA-DQB1*, *HLA-DPA1*, *HLA-DPB1* and *HLA-DRB1* at a two-field resolution. The T1DGC reference panel can be obtained from the NIDDK repository at https://www.niddkrepository.org/niddk/home.do. This panel was the reference also used in previous studies [13], and it will allow for an easier comparison with state-of-the-art tools. We used this panel as provided originally with the SNP2HLA package.

As a testing panel for our imputation method, we used the British 1958 Birth Cohort (1958BC) [19] composed of 2434 individuals genotyped on Illumina HumanHap550 and also typed by gold-standard methods at two-field or one-field levels for *HLA-A*, *HLA-B*, *HLA-C*, *HLA-DQB1* and *HLA-DRB1*. Access to this data was obtained through the Wellcome Trust Case Control Consortium Data Access Committee and could be done from the European Genome-phenome Archive (EGA) at https://www.ebi.ac.uk/ega/. 1958BC was also used as a testing panel in previous studies [13].

These panels cover a variety of existing common alleles in European population: for 1958BC and T1DGC respectively, we have 25 and 51 alleles for *HLA-A*, 44 and 98 for *HLA-B*, 21 and 34 for *HLA-C*, 34 and 52 for *HLA-DRB1*, and 18 and 19 for *HLA-DQB1*. The method used in these panels to attribute HLA alleles has since their publication been shown to cause some systematic errors, for instance HLA-DRB1*14:54 vs DRB1*14:01:01 in [20].

We have also used the panel of 5008 haplotypes from various origins [21], assembled by the 1000 Genomes project in which the SNP/indels were phased thanks to the ShapeIT software [22]. This panel was required to extend (by imputation with IMPUTE2 [23]) the SNP coverage of genotypes obtained by chips into the HLA exonic regions, since this step is needed for HLA-check.

We also used the HLA reference database, called IPD-IMGT/HLA Database (http://hla.alleles.org/), version 3.22 (Oct 2015). This database defines at all levels (protein, cDNA, and gDNA) all the known HLA alleles.

### HLA typing by imputation

To impute HLA from SNP data, we included all HLA alleles, each of which was represented as a biallelic SNP marker: present or absent. We used the ShapeIT software to phase haplotypes, and IMPUTE2 software to impute the HLA alleles. Then, we kept only alleles with a post-probability dosage of more than 0.5, thus defining the individuals for which HLA was “called”. In 99.9% of the cases this indeed gave us exactly two alleles.

### Measure of the accuracy of HLA imputation

HLA-check checks if an HLA allele attribution is compatible with the given SNP genotype of an individual. To assess the accuracy of HLA-check and to measure its efficiency, we imputed the HLA of 2434 individuals in the testing panel (1958BC) with ShapeIT and Impute2 as described above. We measured accuracy as the fraction of correctly assigned HLA allele over all called alleles (i.e., discarding alleles with post-probability dosage less than 0.5). We used such a measure keeping in mind the potential applications: indeed, if we impute HLA alleles for an individual, we will be first interested in seeing if there is a called result, second in knowing if this result is actually correct.

### HLA-check: the approach

The principle of the method is to compare the SNP genotypes of an individual obtained by any experimental (i.e. chip) or computational method (i.e. imputation) with the SNP genotypes defined by all the known combinations of HLA alleles from the IPD-IMGT/HLA Database (http://hla.alleles.org). We then check if the attributed HLA allele pair is among the best matches by computing a discrepancy measure *D*.

Our approach is straightforward: we first try to impute as many SNPs as possible in the HLA exonic regions, using the best reference set at our disposal. As of today, the 1000 genomes project has the best coverage of SNPs with a large panel of already phased genotypes from various populations all over the genome, but similar results will likely be obtained with the Haplotype Reference Consortium [24] in the near future. This SNP extension phase needs to be done as precisely as possible, in order to get a coverage as precise and as complete as possible in the exonic sequences of the HLA genes.

For an individual, for each SNP in the exonic segments of a given HLA gene, we then compare the post-probabilities genotype obtained after its imputation with the genotypes from all possible allele pairs derived from the HLA reference genome, obtained through the available exonic HLA cDNA sequences of IPD-IMGT/HLA Database (http://hla.alleles.org). We do a per-SNP evaluation of the discrepancy measure where *D*, for a pair of HLA alleles, at a given SNP marker, is the probability of this HLA pair to be incorrect. For instance, take rs41541913. If in the HLA definition, HLA-A*01:01 has a guanine (G nucleotide) and HLA-A*80:01 has an cytosine (C nucleotide), and the posterior probabilities for the tested individual are 10%CC, 25%GG and 65%CG, we consider that the imputation post-probabilities indicate *s*=0.35 of disagreement with the (HLA-A*01:01, HLA-A*80:01) HLA allele pair.

Then, *D* obtained for an HLA allele combination for a given individual is simply obtained by summing it for all SNP markers available in the gene^1^:

$$D(genome, HLA_{1}, HLA_{2}) = \sum_{rs}D(rs) $$

After computing *D* for all the pairs of HLA alleles of the IPD-IMGT/HLA Database, we compare the discrepancy measure of the attributed HLA of an individual with the best one obtained among all HLA pairs tested: the final *D* we compute is simply the difference between those. A difference of 0 indicates that we reached the least possible discrepancy for the attributed HLA allele pair, making us highly confident in the validity of this HLA typing, while a high discrepancy indicates a mismatch between the best possible HLA fitting with the genotype and the attributed HLA, suggesting that these HLA alleles are unlikely to be well attributed.

Interestingly, our approach depends mainly on the definitions of the HLA alleles from the IPD-IMGT/HLA Database. All HLA allele combinations are tested, and only the quality of imputation in the HLA exons and the SNP coverage will have an impact.

## Results

### Replication of SNP2HLA results with ShapeIT and IMPUTE2

We first replicated the SNP2HLA method for imputation of HLA alleles, by phasing the same reference panel with the ShapeIT [22] software developed by our group and by imputing SNPs and HLA in the region with the IMPUTE2 software. The results were quite similar to those obtained by SNP2HLA for the quality of imputation (Table 1).

**Table 1:** Comparison of imputation scores using vanilla SNP2HLA vs our method: the results are quite similar

HLA (two-field)	Test population	*D* (ShapeIT/Impute2)	*D* (SNP2HLA)
*HLA-A*	865	97.6	97.1
*HLA-B*	1495	96	94.9
*HLA-C*	813	95.8	95.9
*HLA-DRB1*	800	87.9	87.8
*HLA-DQB1*	974	94.8	96.4

### HLA-check: detection of spurious alleles

**Fig. 1:**
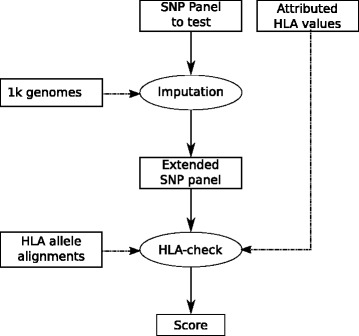
HLA-check pipeline: We start by augmenting the panel to test to get more SNP using an imputation phase with the 1000genomes data, then we compare those SNP alleles with their theoretical values for all possible HLA allele pairs

We also developed a tool, HLA-check, to detect spurious attributed HLA alleles, as described in Material and Methods. This tool relies both on the precise SNP imputation in the exonic parts of HLA genes currently using the 1000 genome reference panel and on the genetic description in the exonic regions of all known HLA allele pairs using the HLA reference database (see pipeline Fig. 1).

**Fig. 2:**
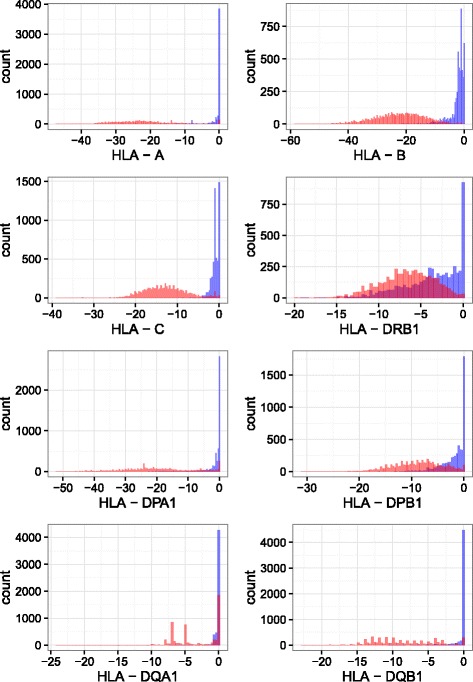
Histogram of HLA-check distance (*D*) distribution obtained for each HLA locus (*A, B, C, DRB, DPA, DPB, DQA, DQB*) comparing the actual T1DGC HLA types (*blue*) with a randomized set of HLA types from T1DGC (*red*). In the x-axis, the *D* value, in the y-axis, the number of subjects (genotypes) obtaining this value. For each SNP genotype, *D* was computed as described in the Material and Methods. One can see the clear-cut difference between the two distributions for class I HLA genes

We first evaluated the soundness of HLA-check on the T1DGC reference panel that contains 5225 individuals typed at a two-field resolution, who were also genotyped using an Illumina chip. For that, we compared the discrepancy measures given by HLA-check for the HLA alleles attributed to the T1DGC individuals with those obtained in randomized tests in which the HLA assignments were shuffled between individuals. Figure 2 presents the curves obtained for *HLA-A*, *HLA-B*, *HLA-C*, *HLA-DRB1*, *HLA-DPA1*, *HLA-DPB1*, *HLA-DQA1*, *HLA-DQB1* in the original and in the shuffled T1DGC population. Several randomizations of the T1DGC population were tested with identical results.

As expected, we observed a clear-cut difference between the distribution of *D* for randomized HLA types and the distribution of *D* for the real population at almost all loci, except for *DRB1*. In *DRB1*, the genotyping by chip is likely of poorer quality since there are several paralogous sequences in the neighborhood (genes *DRB3*, *DRB4*, *DRB5* and pseudo-genes *DRB2*, *DRB6*, *DRB7*, *DRB8*, *DRB9*) and there are also fewer SNPs in the exonic parts of the gene (3 times less than for HLA class 1 genes for instance, cf Table 2). This has also been observed in other works, for instance in HLA*PRG [25] that takes into account paralogous sequences to reach a decent HLA typing rate (from NGS sequences) while observing high similarity between *HLA-DRB1,DRB3,DRB4* and *DRB5*, or in [26].

**Table 2:** Number of SNP markers used for each HLA gene (exonic SNPs that can be imputed from 1000genomes)

HLA	*HLA-A*	*HLA-B*	*HLA-C*	*HLA-DRB1*	*HLA-DPA1*	*HLA-DPB1*	*HLA-DQA1*	*HLA-DQB1*
#snps	118	118	110	41	46	42	71	74

This precludes the use of HLA-check for *HLA-DRB1* and explains the unsatisfactory overlap observed in *D* distributions. In *HLA-DQA1*, the small numbers of bars is due to the very small number of alleles. To choose the cutoff value, we modeled the distribution of *D* attributed to random HLA alleles as a normal distribution and discarded those inferior to *μ*+2*σ* where *μ* is the mean value and *σ* the standard deviation. The chosen values for the cutoff were then rounded to 2 for *HLA-A* and *HLA-C*, and to 7 for *HLA-B*. This result shows it is possible to discriminate discrepancy measures likely corresponding to a plausible HLA allele attribution from the ones corresponding to an aberrant one.

### Impact of the cleaning of the reference panel on imputation quality

We first thought it was possible to identify aberrant HLA attribution from reference panels and delete them for future HLA imputation studies. We indeed tried this approach on 1958BC using T1DGC as the reference panel, but observed no measurable improvement of the HLA imputation accuracy, likely due to the small numbers of removed individuals at stake. Alternatively, using 1958BC as a reference panel, we see that only in the case of *HLA-C*, which had around 5% of dubious HLA typings (a much higher rate than for other HLA genes), we were able to significantly improve the imputation rate of HLA imputation in the T1DGC panel.

### Trading cohort size for precision in HLA imputation

We then used HLA-check to evaluate the credibility of imputed HLA alleles, our goal being to detect and eliminate subjects whose imputed HLA alleles were deemed unlikely, hence improving the accuracy of the HLA imputation.

The first step was to remove from the test population (1958BC) the few individuals for whom *D* on the real (typed) HLA allele was deemed too high, thus eliminating potential badly typed people from our study. These individuals could have shown up as not imputed properly in the following steps. The number of individuals removed following this initial step are provided for the *HLA-A*, *HLA-B*, and *HLA-C* typed at one-field and two-field (Table 3). *HLA-B* is known to be more difficult to impute and type due to its greater polymorphism and heterogeneity, and we also mirror this observation here by having worse results with it than with other class I loci. We also provide our results for *HLA-DQB1*, even if it does not compare well with the class I HLA genes. We have seen that our approach is not relevant for *HLA-DRB1*.

**Table 3:** Test subjects eliminated a priori from the 1958BC test dataset

HLA	*HLA-A*	*HLA-B*	*HLA-C*	*HLA-DQB1*
1958BC (two-field)	18/865	60/1495	77/813	28/974
1958BC (one-field)	35/1669	61/1562	99/1291	103/1701

We then imputed the HLA alleles at one-field and two-field of the 1958BC population using the reference cohort (T1DGC) two-field HLA alleles, and computed *D* obtained on the imputed HLA. People with too high discrepancy were suspected of having a wrong HLA obtained by the imputation, and were labeled as such. This group was indeed very enriched in wrongly imputed HLA alleles (as compared with their HLA attributed by genotyping), and by categorizing them “dubious” we were able to greatly increase the success rate on the remaining test subjects (Table 4). In that table we also give the error rate (rightly typed individuals filtered out by our method).

**Table 4:** Imputation accuracy without any processing, then with the filtering applied with our scoring method

Gene	Test population	Base imputation	People removed	Sub-population imputation
HLA (two-field)				
*HLA-A*	865	97.6	30 (40% correct)	99.6
*HLA-B*	1495	96	112 (65% correct)	98.5
*HLA-C*	813	95.8	81 (60% correct)	99.5
*HLA-DQB1*	974	94.8	40 (60% correct)	96.1
HLA (one-field)				
*HLA-A*	1669	97.8	62 (40% correct)	99.9
*HLA-B*	1562	97.1	119 (70% correct)	99.5
*HLA-C*	1291	95.8	125 (60% correct)	99.9
*HLA-DQB1*	1701	98.2	175 (90% correct)	99.2

T1DGC was used as our reference panel and 1958BC as our test panel. We also precise the percentage of *correct* imputations that were removed

## Discussion

We have developed a simple method to detect aberrant HLA attribution in individuals knowing their SNP genotype in the MHC region. This method is useful for experimentally typed HLA as well as imputed data. In the experimentally typed cohorts, we found very few obvious errors of allele attribution for all HLA genes in T1DGC (less than 1%), significantly more in 1958BC (Table 3). This difference may be explained by the improvement of typing methods between those two cohorts (T1DGC is much more recent). However, after HLA imputation, we could detect several individuals with falsely attributed HLA, and when removing them thanks to HLA-check, we achieved a score of accuracy around 99% for *HLA-A*, *HLA-B*, and *HLA-C* at two-field on the test group 1958BC. These results are quite satisfactory for class I gene alleles since we gain more than 2.5 points of accuracy compared to the use of SNP2HLA alone (Table 4). HLA-check did not yield as good results for class II HLA genes with only a small improvement of accuracy for *HLA-DQB1* (from 94% to 96%) and no improvement at all for *HLA-DRB1*. This latter gene is known to be difficult for genotyping. *HLA-DPA1* and *HLA-DPB1* data were not available for testing (no data in the testing panel). As expected, the results for 1-field typing are even higher, with at least 99.2% accuracy.

Even if we are able to categorize a group of people containing a higher proportion of wrongly imputed, and found possible to identify precisely extreme individuals with clearly false typing, it is still difficult to detect in the people we remove those with false typing from the others (correct people outlined in Table 4), and thus to provide a general model for the detected discrepancies.

There are other tools for HLA imputation such as HIBAG which exhibited quite good performances [14]. We also tried our method on HIBAG for two-field class I HLA genes, and it gave similar results and improvements (Table 5). Note that this is not directly comparable to the other imputation method since we used a pre-built reference file (this tool gives HLA imputation results much faster than other methods, but the reference data for a given chip needs to be computed and is a very time-consuming process), so we could not control the reference panel or use the same as with SNP2HLA.

**Table 5:** Imputation accuracy for HIBAG. Unlike SNP2HLA, we did not use T1DGC as a reference panel but a precomputed model due to the way HIBAG works. Nevertheless, our results are very similar to those obtained with SNP2HLA

HLA (two-field)	Test population	Base imputation	People removed	Sub-population imputation
*HLA-A*	865	97	36	99.5
*HLA-B*	1495	95	84	97.3
*HLA-C*	813	95	83	99.7

## Conclusion

In the last decade, millions of individuals have been typed through genotyping chips for genetic association studies and the current accuracy of imputation software such as SNP2HLA may limit the statistical power for finding new associations on HLA genes. The use of HLA-check would certainly remove a small proportion of individuals, but could allow a higher accuracy in association detection justifying its use for research purposes. Moreover, these removed individuals could be individually retyped if needed (they are about 5% of typings). To this end, HLA-check can be downloaded for its local use. HLA-check performs very quickly (on a personal computer): only a few seconds per tested individual are needed to obtain the final comparison value for a given HLA. This method should not have a direct impact on HLA typing for medical purposes since current sequencing methods already reach 100% of accuracy at G group [27] level (exons 2 and 3 for class I and exon 2 for class II).

## Availability and requirements

HLA-check is available under the MIT license at url https://github.com/mclegrand/HLA-check/. This license expressly allows for any use or modification for one’s own needs. It is available as a platform-independent C++11 source code, and can be compiled with openMP to enable threading.

## Endnote

^1^ We also tried to compute the sum the log of (1-s) to obtain the combined probability of all markers, but this approach gave a considerable importance to imputation errors and ended up with more imprecise results than the simple sum.

## Abbreviations

1958BC: British 1958 Birth cohort
EGA: European Genome-phenome archive
HLA: Human leukocyte antigen
IPD-IMGT: Immuno Polymorphism Database - International ImMunoGeneTics information system
MHC: Major histocompatibility complex
NIDDK: National [USA] Institute of diabetes and digestive and kidney diseases
NGS: Next generation sequencing
SNP: Single nucleotide polymorphism
SSOP: Sequence-specific oligonucleotide probes
SSP: Sequence-specific primers
T1DGC: Type 1 diabetes genetics consortium
